# Double-stranded RNA analog and type I interferon regulate expression of Trem paired receptors in murine myeloid cells

**DOI:** 10.1186/s12865-016-0147-y

**Published:** 2016-05-03

**Authors:** Jun Kasamatsu, Mengyao Deng, Masahiro Azuma, Kenji Funami, Hiroaki Shime, Hiroyuki Oshiumi, Misako Matsumoto, Masanori Kasahara, Tsukasa Seya

**Affiliations:** Department of Microbiology and Immunology, Hokkaido University Graduate School of Medicine, Kita-15, Nishi-7, Kita-ku, Sapporo, 060-8638 Japan; Department of Pathology I, Hokkaido University Graduate School of Medicine, Kita-15, Nishi-7, Kita-ku, Sapporo, 060-8638 Japan; Present address: Department of Microbiology and Immunology, Washington University School of Medicine, 660 South Euclid Avenue, St. Louis, MO, 63110 USA; Present address: Department of Immunology, Graduate School of Medical Sciences, Kumamoto University, 1-1-1, Honjo, Chuo-ku, Kumamoto, Japan; Department of Vaccine Immunology, Graduate School of Medicine, Hokkaido University, Kita-ku, Sapporo, 060-8638 Japan

**Keywords:** RNA sensors, Paired receptors, Dendritic cells, Macrophages, Type I interferon, Trem family, Evolution

## Abstract

**Background:**

Triggering receptors expressed on myeloid cells (Trem) proteins are a family of cell surface receptors used to control innate immune responses such as proinflammatory cytokine production in mice. Trem genes belong to a rapidly expanding family of receptors that include activating and inhibitory paired-isoforms.

**Results:**

By comparative genomic analysis, we found that Trem4, Trem5 and Trem-like transcript-6 (Treml6) genes typically paired receptors. These paired Trem genes were murine-specific and originated from an immunoreceptor tyrosine-based inhibition motif (ITIM)-containing gene. Treml6 encoded ITIM, whereas Trem4 and Trem5 lacked the ITIM but possessed positively-charged residues to associate with DNAX activating protein of 12 kDa (DAP12). DAP12 was directly associated with Trem4 and Trem5, and DAP12 coupling was mandatory for their expression on the cell surface. In bone marrow-derived dendritic cells (BMDCs) and macrophages (BMDMs), and splenic DC subsets, polyinosinic-polycytidylic acid (polyI:C) followed by type I interferon (IFN) production induced Trem4 and Treml6 whereas polyI:C or other TLR agonists failed to induce the expression of Trem5. PolyI:C induced Treml6 and Trem4 more efficiently in BMDMs than BMDCs. Treml6 was more potentially up-regulated in conventional DC (cDCs) and plasmacytoid DC (pDCs) than Trem4 in mice upon in vivo stimulation with polyI:C.

**Discussion:**

Treml6-dependent inhibitory signal would be dominant in viral infection compared to resting state. Though no direct ligands of these Trem receptors have been determined, the results infer that a set of Trem receptors are up-regulated in response to viral RNA to regulate myeloid cell activation through modulation of DAP12-associated Trem4 and ITIM-containing Treml6.

**Electronic supplementary material:**

The online version of this article (doi:10.1186/s12865-016-0147-y) contains supplementary material, which is available to authorized users.

## Background

Some multiple immune receptors, such as signal regulatory protein (SIRP), CD200R, killer inhibitory receptor (KIR), lymphocyte antigen 49 (Ly49), CD300, have a highly homologous extracellular domain [1–4]. These receptors are termed “paired receptors” that are generally defined by the following characteristics: 1. they have a high level of amino acid sequence identity within their extracellular domains, 2. they are encoded by different genes, but located in the same gene cluster, 3. they have both activating receptor associated by immunoreceptor tyrosine-based activation motif (ITAM)-containing adaptor, such as DNAX activating protein of 12 kDa (DAP12), and immunoreceptor tyrosine-based inhibition motif (ITIM)-containing inhibitory receptors [4–6]. Their activating and inhibitory receptors recognize endogenous/exogenous components and often differ in their binding specificity. Paired receptors expressed on myeloid cells including dendritic cell (DC) subsets have been poorly understood in their functional features concerning innate-adaptive interplay, whereas paired NK receptors including Ly49, KIR, and Natural Killer group 2 (NKG2)/CD94, have been characterized to explain the “missing self” activity of NK cells [3, 7]. Therefore, we attempted to investigate the role of uncharacterized paired receptors in myeloid cells. We first searched for genes of myeloid paired receptors in mammalian genome database and analyzed their expression profiling and immune-related function.

Triggering receptors expressed on myeloid cells (Trem) proteins are a family of cell surface receptors expressed broadly on myeloid cells [8]. The receptors are a structurally-related protein family that generally contain a single immunoglobulin (Ig) domain in the extracellular region, and their gene cluster is located on mouse chromosome 17C and human chromosome 6p21.1 [9, 10]. Inhibitory Trem proteins, containing cytoplasmic ITIM recruit protein tyrosine phosphatases such as Src homology 2 domain-containing phosphatase 1 (SHP-1) and SHP-2 [11]. On the other hand, activating Trem proteins lack cytoplasmic signaling elements, but contain a charged residue in their transmembrane domain that facilitates the association with DAP12, which functions as docking sites for Syk tyrosine kinases that mediate PLCγ and PI3K activation [12, 13].

Trem and Trem-like genes such as novel immune type receptors have also been reported in teleost [14–16]. Human Trem1 is an activating receptor expressed at high levels on neutrophils and monocytes that infiltrate human tissues infected with bacteria [17]. Recent studies have shown that Trem1 has a critical role in negative regulation of proinflammatory cytokine production in vivo [18, 19]. Trem2 is also activating receptor expressed on DCs, osteoclasts and microglia and control not only immune function such as maturation of DCs but also osteoclastogenesis [20, 21]. Functional DAP12-associated Trem3 is present in mice, but not primates [22]. Treml4 binds to dead cells and accelerates antigen presentation and priming of CD4^+^ and CD8^+^ T cells in vivo [23, 24]. Trem4 (also known as pDC-Trem) is involved in IFN-α production from Fms-related tyrosine kinase 3 (Flt3) ligand-induced plasmacytoid DCs (pDC), and TLR signals are essential for Trem4 expression [25]. Nevertheless, other Trem and Treml receptors have been poorly characterized in mice, although they show myeloid cell-dominant expression [8]. There is so far no report on the comprehensive analysis of the mouse Trem/Treml gene cluster, and we found paired receptor-like signatures in several Trem receptors on database search.

In this study, we focused on orphan and uncharacterized Trem genes, named murine Trem4 (deposited as pDC-Trem [25]), Trem5 (known as 9830107B12Rik) and Treml6 (known as B430306N03Rik), and elucidated their evolutional stands in vertebrates. The ligands of these receptors remain undetermined, but we noticed that polyI:C signal dynamically up-regulated the Trem4 and Treml6. Although the extracellular immunoglobulin (Ig) domain of Trem4, Trem5 and Treml6 were closely related to each other, Treml6 encoded ITIM in its cytoplasmic region, whereas Trem4 and Trem5 lacked ITIM but had positively-charged residues to associate with DAP12. Together, these expression features indicate that these receptors are typical paired receptors, which modulate innate signal in myeloid cells like the case of Trem1 and Trem2 [26]. Trem4 and Treml6 may be up-regulated on the cell-surface in myeloid cells by viral RNA to counteract or modulate presetting immune response.

## Methods

### Mice

All mice were backcrossed with C57BL/6 mice more than seven times before use. *Ifnar1*^−/−^ mice were obtained from the Jackson Laboratory. *Ticam-1*^−/−^ and *Mavs*^−/−^ mice were generated in our laboratory [27, 28]. All mice were maintained under specific pathogen-free conditions in the animal facility of the Hokkaido University Graduate School of Medicine. Female mice 8–12 weeks of age were used in all experiments, all of which were performed according to the guidelines issued by Hokkaido University Animal Care and Use Committee, who approved this study as ID number: 08–0243, “Analysis of immune modulation by toll-like receptors”. Mice were sacrificed according to the rule of animal euthanasia. Animal experiments protocols and guidelines were approved by the Animal Safety Center, Hokkaido University, Japan.

### Cells

Bone marrow-derived dendritic cells (BMDCs) were generated by culturing bone-marrow cells for 6 days in RPMI 1640 (GIBCO Invitrogen GmbH, Karlsruhe, Germany) with 10 % heat-inactivated FBS containing GM-CSF (10 ng/ml, Peprotech, Rocky Hills, NJ, USA). Bone marrow-derived macrophages (BMDMs) were prepared by culturing bone-marrow cells in RPMI 1640 for 6 days with 10 % heat-inactivated FBS containing 30 % L929 supernatant. Concentrations of stimulants used were as follows: Pam3CSK4 (Roche, Indianapolis, IN, USA), 5 μg/ml; polyI:C (GE Healthcare Biosciences, Uppsala, Sweden), 20 μg/ml; LPS (Sigma–Aldrich, St. Louis, MO, USA), 0.1 μg/ml; Flagellin A (FlaA), 0.3 μg/ml; CpG (M362, Invivogen, San Diego, CA, USA), 2.5 μg/ml with DOTAP (Roche, Indianapolis, IN, USA). FlaA was synthesized in our previous study. Wild-type, *Ticam-1*^−/−^, *Mavs*^−/−^ and *Ifnar1*^−/−^ BMDCs were stimulated with polyI:C (20 μg/ml). For blocking experiment of type I IFN receptor, anti-IFN receptor-neutralizing antibody (Biolegend, San Diego, CA, USA) or isotype control　antibody (Biolegend, San Diego, CA, USA) were added at a final concentration of 10 μg/ml. HEK293FT cells were maintained in DMEM (GIBCO Invitrogen GmbH, Karlsruhe, Germany) supplemented with 10 % heat-inactivated FBS and antibiotics. For isolation of cDCs and pDCs, spleens were treated with 400 IU Mandle U/ml collagenase D (Roche, Indianapolis, IN, USA) at 37 °C for 25 min in HBSS (Sigma–Aldrich, St. Louis, MO, USA). EDTA was added, and the cell suspension was incubated for an additional 5 min at 37 °C. After removal of red blood cells with ammonium chloride–potassium lysis buffer, CD11c^+^ DCs were isolated using CD11c and PDCA-1 MACS beads (Miltenyi Biotec, Auburn, CA, USA). MACS-sorted DCs were stained with anti-B220–FITC (RA3-6B2, eBiosciences, San Diego, CA, USA) and anti-CD11c–APC (N418, Biolegend, San Diego, CA, USA) and CD11c^+^/B220^−^ cDCs and CD11c^+^/B220^+^ pDCs were sorted by using FACS Aria II (BD Biosciences, San Jose, CA, USA). The purity of sorted cells was > 98 %.

### Database analysis

A BLAST search of the vertebrate Trem genes was performed by the amino acid sequences of human and mouse Trem genes as queries using the Ensembl genome databases (http://www.ensembl.org/index.html). The genome assembly versions of species used in this paper were as follows: GRCh37 of human, GRCm38 of mouse, OryCun2.0 of Rabbit, CanFam3.1 of Dog, EquCab2 of Horse, UMD3.1 of Cow, Sscrofa10.2 of Pig, BROADO5 of opossum, Galgal4 of chicken, JGI_4.2 of frog and Zv9 of zebrafish. Domain structures of Trem proteins were analyzed by SMART program (http://smart.embl-heidelberg.de). A phylogenetic tree based on the amino acid sequences of Ig domain was constructed by the Neighbor-joining (NJ) method in the ClustalX version 2 program and the MEGA version 5 program [29, 30]. The distance matrix was obtained by calculating p-distances for all pairs of sequences. Sites containing gaps were excluded from the analysis using the pairwise deletion option. The reliability of branching patterns was assessed by bootstrap analysis (1000 replications). Vertebrate Trem genes used for phylogenetic and comparative genomic analysis are listed in supplementary Table 1.

**Table 1:** Identity matrix comparison of Ig domains in murine Trem proteins

	Trem5	Treml6	Trem4
Trem5	—	—	—
Treml6	80.4 %	—	—
Trem4	78.4 %	88.7 %	—
Trem1	27.3 %	23.9 %	26.4 %
Trem2	23.9 %	23.9 %	24.8 %
Trem3	22.2 %	22.4 %	23.1 %
Treml1	32.7 %	21.9 %	24.3 %
Treml2	24.8 %	23.2 %	25.7 %
Treml4	34.0 %	32.1 %	34.0 %

### RNA isolation and cDNA synthesis

Total RNA from BMDCs, BMDMs, splenic DC subsets, and tissues was extracted using TRIzol reagent (Invitrogen, Carlsbad, CA, USA), after which 0.1–1 mg RNA was reverse transcribed using a high-capacity cDNA transcription kit with an RNase inhibitor kit (Applied Biosystems Foster City, CA, USA), according to the manufacturer’s instructions.

### Gene cloning and expression vectors

Murine Treml6/Trem4/DAP12 genes and Trem5 gene were amplified by KOD-plus DNA polymerase (Toyobo, Osaka, Japan) from polyI:C-stimulated BMDCs and unstimulated spleen cDNA, respectively. The primers for the Trem5 gene: 5′-GTGGTGGCTCCTGAATCC-3′ (forward) and 5′-CTCAGCTCCTCCTTTGACATAGACT-3′ (reverse), Treml6 gene: 5′-TGCCCAGCTTCTCTCTCATC-3′ (forward) and 5′-TAGGAAGTTGCCCCTCCACA-3′ (reverse), Trem4 gene: 5′-TGCCCAGCTTCTCTCTCATC-3′ (forward) and 5′-AGCGAACCCTGAGTAATACAGTCAG-3′ (reverse), Dap12 gene: 5′-GCCCCTGGACTGTGGTGT-3′ (forward) and 5′-GATCCCAGAGAGGGCTTGTT-3′ (reverse). The open reading frame (ORF) sequences of Trem5, Treml6, Trem4 and Dap12 genes were completely conformed to reported sequences of NM_001177896, NM_177083, AB372005 and NM_011662, respectively. These cDNA encoding the full-length ORF was cloned into a pEF-BOS multi-cloning site, and an HA or FLAG sequence was inserted behind signal peptide sequence of paired Trem and DAP12 genes, respectively [31]. The plasmids were sequenced to confirm that there were no PCR errors.

### Immunoblotting and immunoprecipitation

HEK293FT cells were transfected in 6-well plates with plasmids encoding FLAG-tagged DAP12 and/or HA-tagged paired Trem genes by using Lipofectamine 2000 (Invitrogen, Carlsbad, CA, USA). The plasmid amounts were normalized by the addition of empty plasmid. At 24 h after transfection, cells were lysed with lysis buffer (20 mM Tris–HCl [pH 7.5], 150 mM NaCl, 1 mM EDTA, 10 % glycerol, 1 % Nonidet P-40, 30 mM NaF, 5 mM Na3VO4, 20 mM iodoacetamide, 2 mM phenylmethylsulfonyl fluoride), and then proteins were immunoprecipitated with mouse anti-HA monoclonal antibody (Sigma–Aldrich, St. Louis, MO, USA). The lysates and precipitates were analyzed by SDS-polyacrylamide gel electrophoresis (SDS-PAGE) followed by immunoblotting with rabbit anti-HA polyclonal antibody (Sigma–Aldrich, St. Louis, MO, USA) or mouse anti-FLAG M2 monoclonal antibody (Sigma–Aldrich, St. Louis, MO, USA).

### Flow cytometric analysis

At 24 h after transfection, transfected cells were incubated with FACS buffer (DPBS containing 0.1 % BSA and 0.1 % sodium azide) containing 10 % goat serum (Cedarlane, Ontario, Canada) on ice. After incubation, the cells were washed twice with FACS buffer and labeled with anti-HA polyclonal antibody and anti-FLAG M2 monoclonal antibody for 30 min on ice. After washing, anti-mouse IgG fluorescein (FITC) labeled secondary antibody (American Qualex, San Clemente, CA) and anti-rabbit IgG phycoerythrin (PE)-labeled secondary antibody (Biolegend, San Diego, CA, USA) was added and the cells were further incubated for 30 min on ice. Stained cells were then analyzed using a FACS Calibur flow cytometer (BD Biosciences, San Jose, CA, USA).

### ELISA

BMDCs (3–5 × 10^5^) seeded on 24-well plates were collected and analyzed for cytokine levels with enzyme-linked immunosorbent assay (ELISA). ELISA kits for mouse IFN-α and IFN-β were purchased from PBL Biomedical Laboratories.

### Quantitative real-time PCR

Quantitative real-time PCR (qPCR) was performed using the Step One Real-Time PCR system (Applied Biosystems, Foster City, CA, USA). The primers for Trem5 gene: 5′-ATTCAAGTGCTCCTGCCAAT-3′ (forward) and 5′-AATGGAGATCGGGTCCTGT-3′ (reverse), Treml6 gene: 5′-CAGCCCCGTCACTAACAGAA-3′ (forward) and 5′-CGAATGTGAGCAGTAGCAGAA-3′ (reverse), Trem4 gene: 5′-CCACTGTTGTTTCCTCTCCTGA-3′ (forward) and 5′-AGTCCACAGACCACGGGAAT-3′ (reverse), and *Gapdh:* 5′- GCCTGGAGAAACCTGCCA −3′ (forward) and 5′- CCCTCAGATGCCTGCTTCA −3′ (reverse).

## Results

### Identification of murine paired trem genes

**Fig. 1:**
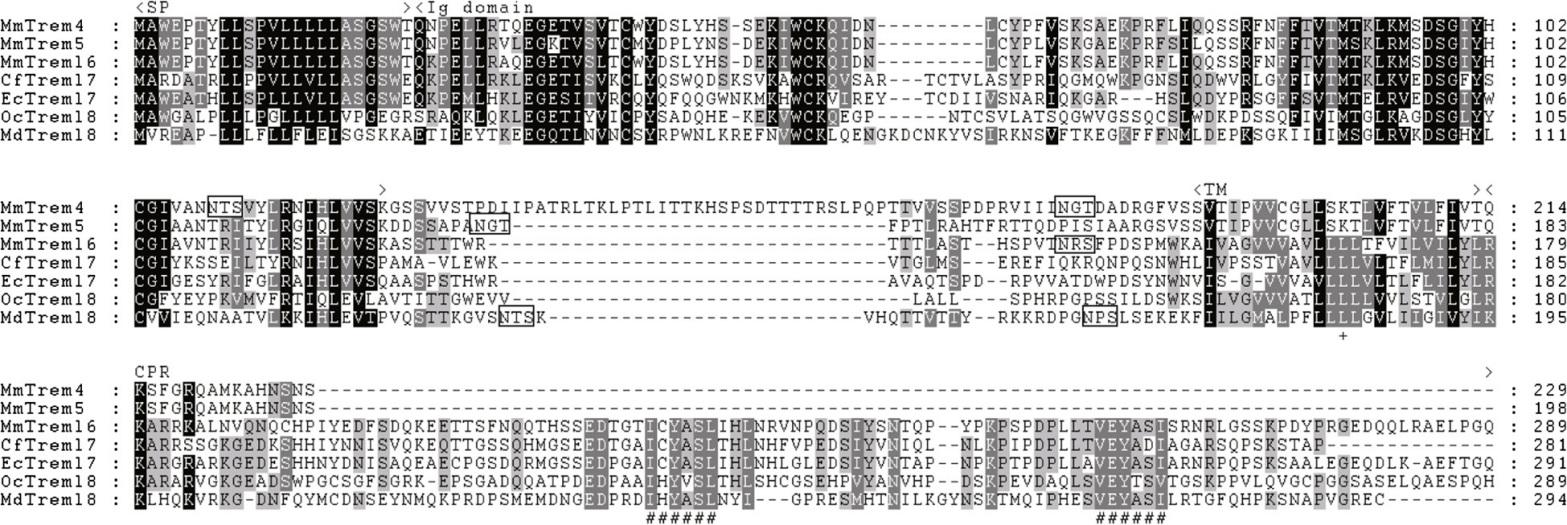
Deduced amino acid sequences of murine paired Trems, Treml7 and Treml8. Murine paired Trems and other mammalian Treml7 and Treml8 are aligned. Boxs indicate potential N-linked glycosylation sites predicted by NetNGlyc program (http://www.cbs.dtu.dk/services/NetNGlyc/). ‘#’ represents the ITIM, defined as (I/V/L/S)-X-Y-X-X-(L/V). ‘+’ represents the lysine residue in the transmembrane region (TM), which is presumed to be involved in the interaction with DAP12. SP, signal peptide; Ig, immunoglobulin; CPR, cytoplasmic region. Black shaded area, 100–90 % identity; gray shaded area, 89–70 % identity; light gray shaded area, 69–50 % identity. Mm; mouse (*Mus musculus*), Oc; rabbit (*Oryctolagus cuniculus*), Cf; dog (*Canis lupus familiaris*), Ec; horse (*Equus caballus*)

All murine Trem proteins are type-I transmembrane proteins containing single extracellular Ig domains [10]. To assess the homology of these Trem proteins, we analyzed amino acid sequence identities of the murine Trem proteins (Table 1). Trem4 was related most closely to Trem5 and Treml6 with high similarity (78.4 % and 88.7 % amino acid identities, respectively). Similarly, the Ig domains of Trem5 and Treml6 showed 80.4 % amino acid identities. On the other hand, the amino acid sequence identities in the corresponding domains of the other Trem genes were 22.2-34.0 %. The deduced Trem4, Trem5 and Treml6 proteins were made up of 198, 229 and 289 amino acids, respectively (Fig. 1). Trem4, Trem5 and Treml6 all encoded a threonine-riched stalk between the Ig domain and transmembrane region (TM), suggesting that they recognize a similar signature of the ligand. Treml6 encoded two ITIMs that formed a conserved sequence of amino acids (S/I/V/LxYxxI/V/L) in the cytoplasmic region (CPR), whereas Trem4 and Trem5 lacking the ITIM of positively-charged residues to enable them to associate with DAP12 in the TM. Consequently, these features indicate that these Trem proteins are typical paired receptors.

### Murine paired trem genes are murine-specific and related most closely to ITIM-containing Treml7 genes

**Fig. 2:**
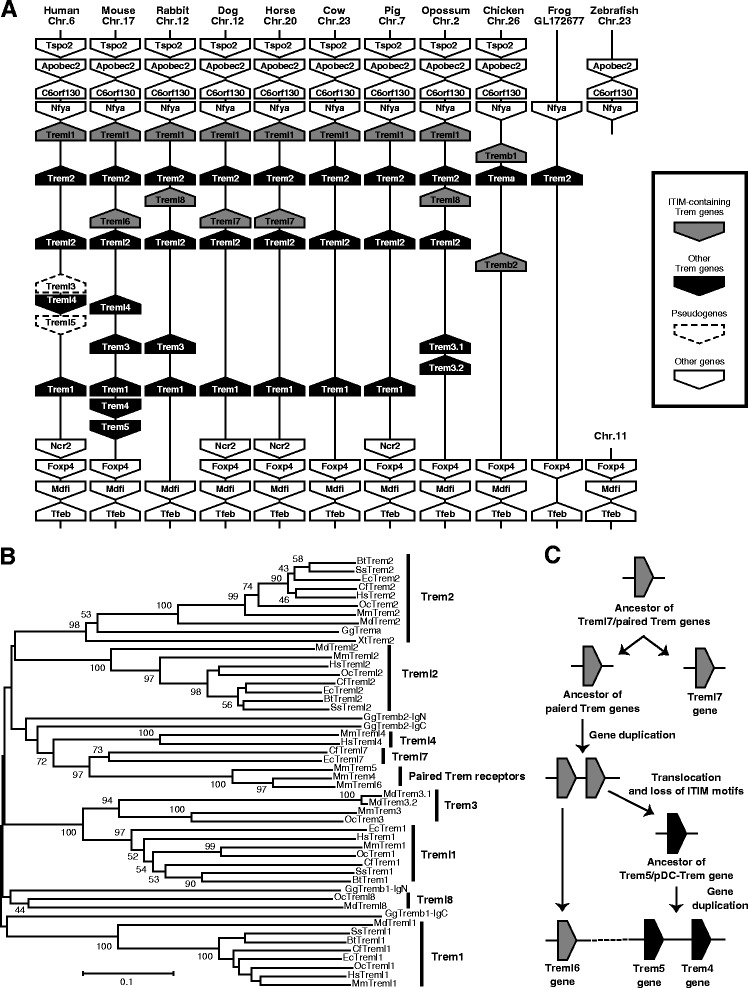
Comparative genome analysis of Trem genes in vertebrates. **a** Synteny block including Trem and their neighbor genes. The arrows represent the genes and their transcriptional orientations annotated in the Ensembl genome data for each species. Gray and black arrows indicate ITIM-containing Trem and other Trem genes, respectively. This figure is not drawn to scale. **b** Neighbor-joining tree of vertebrate Trem genes based on extracellular Ig domains. Numbers at the nodes represent the bootstrap confidence level in percentage. Only bootstrap values over 40 are indicated. Hs; human (*Homo sapiens*), Mm; mouse (*Mus musculus*), Oc; rabbit (*Oryctolagus cuniculus*), Cf; dog (*Canis lupus familiaris*), Ec; horse (*Equus caballus*), Bt; cow (*Bos taurus*), Ss; pig (*Sus scrofa*), Md; opossum (*Monodelphis domestica*), Gg; chicken (*Gallus gallus*), Xt; frog (*Xenopus tropicalis*). **c** Evolutionary model of murine paired Trem genes. Firstly, ancestral paired Trem gene and Treml7 rose from common ancestral gene that encored two ITIMs in its CPR. After brunching to murine linage, ancestral paired Trem gene containing ITIMs were duplicated, and the one of them evolved murine Treml6 gene. Another duplicated gene was translocated between Trem1 and Foxp4 genes and lost ITIMs. Finally, the common ancestor of Trem5 and Trem4 genes was duplicated, and evolved murine Trem5 and Trem4

In BLAST search of the jawed vertebrate genome data deposited in Ensembl genome databases, we found additional two ITIM-containing Trem genes, named Treml7 and Treml8 and identified in dog/horse and rabbit/opossum, respectively (Fig. 1, Additional file 1: Table S1). Their amino acid sequences indicated the presence of two ITIMs in putative CPR of Treml7 and Treml8. To clarify the evolutional history of the vertebrate Trem gene cluster, we performed comparative genome analysis to compare the human and murine Trem genomic regions with the corresponding regions of the rabbit, dog, horse, cow, pig, opossum, chicken, frog (*X. tropicalis*) and teleost (zebrafish). Our BLAST search of the genome data failed to identify obvious Trem genes in the teleost genome, whereas we found a single Trem gene showing high similarity to vertebrate Trem2 in the *X. tropicalis* genome (Fig. 2a). The tetrapod Trem genes are located between the Nfya and Foxp4 genes, and these syntenic blocks are highly conserved between the human and frog genomes. The Treml6, Treml7 and Treml8 genes are all located between the mammalian Trem2 and Treml2 genes. On the other hand, the Trem4 and Trem5 genes are located between the murine Trem1 and Foxp4 genes. To address the relationship between the paired Trem genes and other Trem family members, we constructed a neighbor-joining tree (Fig. 2b). We found that three paired Trem genes formed a unique cluster independent of other vertebrate Trem genes. Hence, murine paired Trem genes are murine-specific. Additionally, these paired Trem genes are related most closely to dog and horse ITIM-containing Treml7 genes by a high bootstrap value. On the other hand, Treml8 genes formed a unique cluster separate from Treml7 and paired Trem genes. A possible process of gene-clustering of the Trem family is depicted in Fig. 2c.

### Trem4 and Trem5 associated with DAP12

**Fig. 3:**
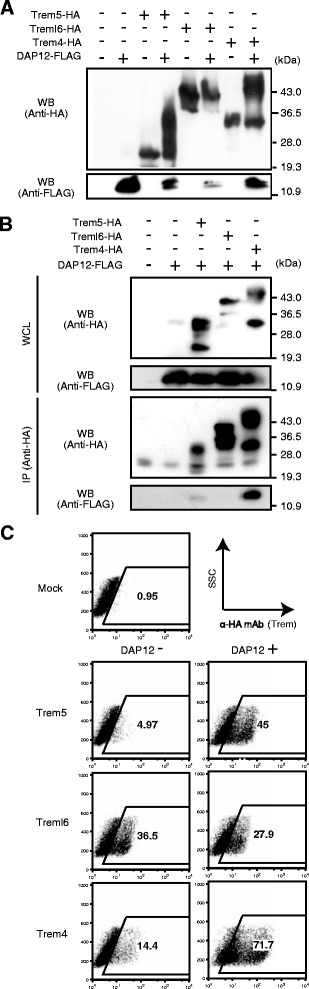
DAP12 binds Trem5 and Trem4 and facilitates Trem’s post-translational modification and cell-surface expression. **a** DAP12 is required for the post-translational modification of Trem5 and Trem4. Whole-cell lysates (WCL) prepared from HEK293FT cells transfected with the indicated combinations of HA-paired Trem proteins and/or FLAG-DAP12 were analyzed by immunoblotting with anti-FLAG or anti-HA Abs. **b** Association of DAP12 with paired Trem proteins. WCL prepared from HEK293FT cells transfected with the indicated combinations of HA-paired Trem proteins and FLAG-DAP12 were immunoprecipitated with anti-HA Ab and detected with anti-FLAG or anti-HA Abs. **c** DAP12 is required for cell surface expression of Trem5 and Trem4. Paired Trems were transfected into HEK293FT cells with or without DAP12. HA-paired Trem proteins on HEK293FT cells were analyzed using anti-HA mAb by FACS

DAP12 is directly associated with Trem4 (pDC-Trem) [25]. This association is essential for expression of Trem4 on the cell surface and its signal transduction. In addition, association of DAP12 with immune receptors is required for post-translational modification including glycosylation in the Golgi apparatus [32]. We predicted the N-glycosylation sites of paired Trem receptors using NetNGlyc program (http://www.cbs.dtu.dk/services/NetNGlyc/), and subsequently identified several sites in the amino acid sequences of these Trem proteins (Fig. 1). To investigate whether DAP12 is required for post-translational modification of paired Trem proteins, we performed immunoblotting analysis using HEK293FT cells transfected with HA-tagged Trem with/without FLAG-tagged DAP12. Immunoblotting of Treml6 and/or DAP12 lysates showed that two bands, mature (high molecular weight band) and immature (low molecular weight band) Treml6 proteins, were detected regardless of co-transfection of the DAP12 plasmid (Fig. 3a). However, immunoblotting of Trem4 and Trem5 lysates from DAP12-untransfected cells showed that only single-immature Trem4 and Trem5 proteins were detected. In contrast, immunoblotting of the lysates from DAP12-transfected cells detected mature Trem4 and Trem5 proteins, which were heavily glycosylated (Fig. 3a). Hence, DAP12-binding is required for post-translational modification of Trem4 and Trem5, but not Treml6. To investigate the association of Trem proteins with DAP12, various combinations of the HA-tagged Trem and FLAG-tagged DAP12 were transfected into HEK293FT cells and were then subjected to immunoprecipitation analyses. DAP12 was co-immunoprecipitated with Trem4 and Trem5, but not Treml6, indicating that DAP12 binds directly to Trem4 and Trem5 in HEK293 cells (Fig. 3b). To investigate whether DAP12 is essential for the Trem4/5’s expression on the cell surface, we performed FACS analysis of Trem and/or DAP12-transfected HEK293FT cells. The expression of Trem4 and Trem5 on the cell surface was induced by co-transfection with DAP12, whereas Treml6 was constitutively surface-expressed regardless of DAP12 transfection under the same conditions (Fig. 3c). Taken together, these data suggest that association with DAP12 is essentially required for post-translational modification and surface-expression of Trem4 and Trem5 proteins.

### Expression of paired trem receptors in BMDCs and BMDMs

**Fig. 4:**
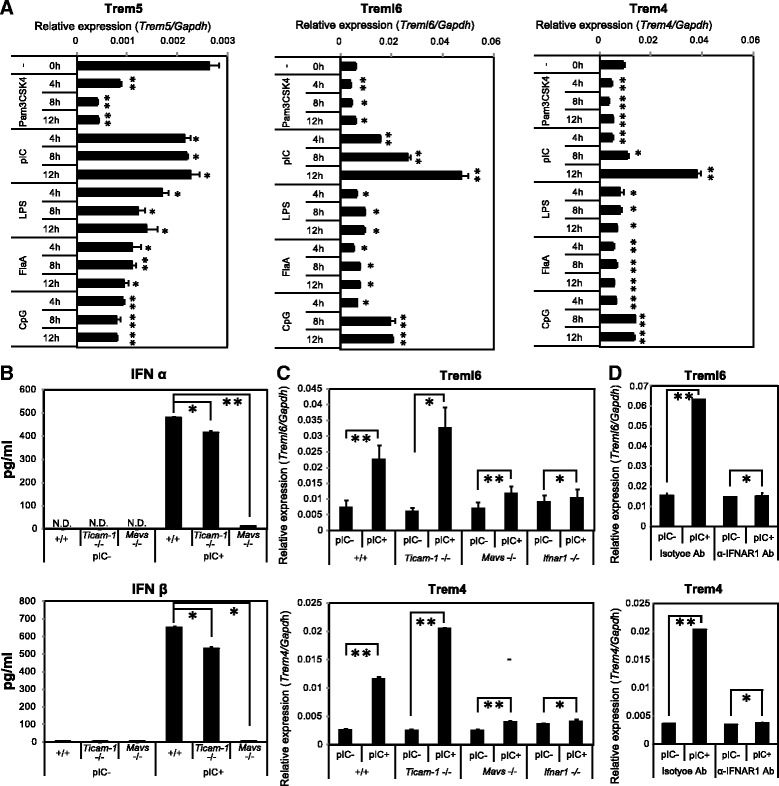
Expression of paired Trem genes in BMDCs. **a** Paired Trems expression after TLR agonist stimulations in BMDCs. Concentrations of stimulants used were as follows: Pam3CSK4, 5 μg/ml; polyI:C (pIC), 20 μg/ml; LPS, 0.1 μg/ml; Flagellin A (FlaA), 0.3 μg/ml; CpG, 2.5 μg/ml with DOTAP. **b** Type I IFN production in Wild-type, *Ticam-1* ^*−/−*^ and *Mavs* ^−/−^ BMDCs. BMDCs prepared from each mouse line were stimulated with pIC (20 μg/ml). 24 h later, the mounts of IFNα and IFNβ in culture supernatants were measured by ELISA. **c** Treml6 and Trem4 mRNA expressions after pIC stimulation in KO mice-derived BMDCs. Wild-type, *Ticam-1*−/−, *Mavs*−/− and *Ifnar1*−/− derived BMDCs were stimulated by pIC (20 μg/ml) for 12 h. **d** Type I IFN signal is indispensable for Treml6 and Trem4 expression of BMDCs derived from Wild-type mice. BMDCs were stimulated with pIC (20 μg/ml) and anti-IFN receptor blocking antibody or isotype control (10 μg/ml) for 12 h. In these experiments, mRNA expressions of paired Trem genes were measured by qPCR and their expression levels were normalized to that of Gapdh mRNA. Data are representative of three independent experiments. The data shown are representative of at least two independent experiments. Data are means ± SD of three independent samples. *p < 0.05. **p < 0.01

**Fig. 5:**
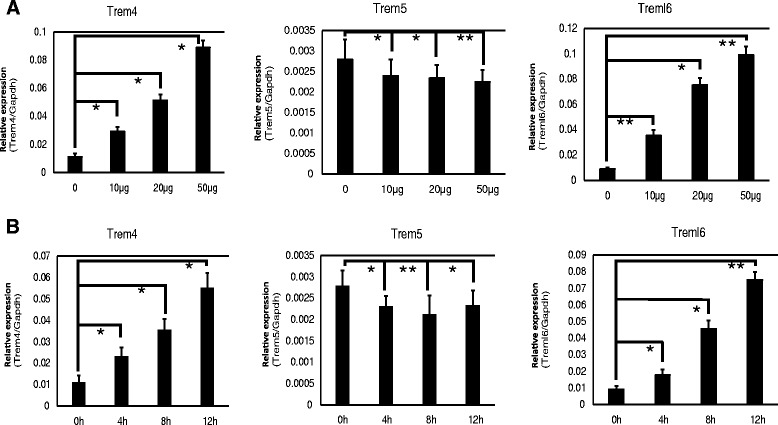
Expression of paired Trem genes in BMDMs. **a**. Dose-dependent expression of paired Trem genes in response to polyI:C stimulation in BMDMs. Day 7-harvested BMDMs (2 × 10^5^ cells /well) were incubated with polyI:C for 12 h, the concentration of polyI:C were 10 μg/ml, 20 μg/ml and 50 μg/ml. **b** Time-dependent expression of paired Trem genes in response to polyI:C stimulation in BMDMs. Day 7-harvested BMDMs (2 × 10^5^ cells /well) were incubated with polyI:C by the concentration of 20 μg/ml. The incubation times with polyI:C were 4, 8 and 12 h. Relative expression levels of Trem genes were evaluated by qPCR. The data shown are representative of at least two independent experiments. Data are means ± SD of three independent samples. *p < 0.05. **p < 0.01

Trem4 expression on pDCs is correlated with TLR-dependent type I IFN production [25]. Treml6 gene expression is induced by type I IFN produced in response to mouse cytomegalovirus infection in CD8α^+^ and CD11b^+^ conventional dendritic cells (cDCs) [33]. Therefore, Trem4 and Treml6 are likely induced via nucleic acids-sensing pathways. To investigate the IFN-inducible pathway for paired Trem gene expression, we performed qPCR analysis for BMDCs stimulated with various TLR agonists. None of the TLR agonists tested stimulated the expression of the Trem5 gene in BMDCs, and Trem5 basal expression was rather decreased in response to TLR2 agonist (Pam3CSK4). In contrast, polyI:C strongly induced the expression of Trem4 and Treml6 genes, ~10-fold over the basal levels (Fig. 4a). Trem-inducing profiles were also observed with macrophages (BMDM) in responses to polyI:C (Fig. 5).

Myeloid cells, macrophages and some DC subsets including cDC play a critical rule in both innate and adaptive immunity. Activation of cDCs by polyI:C robustly promoted the production of type I IFN [34]. In cDCs, both the TLR3/Toll-IL-1 receptor domain-containing adaptor molecule 1 (TICAM-1) and MDA5/mitochondrial antiviral signaling protein (MAVS) pathways lead to production of type I IFN in response to polyI:C [35, 36]. To investigate the role of the TICAM-1 and MAVS pathways in type I IFN production by BMDCs, we stimulated BMDCs derived from *Ticam-1-* or *Mavs*-deficient mice. The results showed that the production of type I IFN was mainly dependent on the MAVS pathway, but not the TICAM-1 pathway (Fig. 4b). To clarify the mechanism of Trem4 and Treml6 induction, we investigated the Trem expression in various genetically-manipulated mice. The expression of Trem4 and Treml6 genes was abolished in BMDCs derived from *Mavs*- and *Ifnar1*-deficient mice (Fig. 4c). Additionally, the expression of the Trem4 and Treml6 genes was completely blocked by treatment with IFNAR1-neutralizing antibody (Fig. 4d). Ultimately, type I IFN signaling is required for the expression of Trem4 and Treml6 in BMDCs. Hence, our data indicate that expression of DAP12-associated Trem4 and Trem5 genes is differentially regulated in BMDCs, whereas Trem4 and Treml6 genes are regulated by polyI:C-induced type I IFN. Likewise, polyI:C-inducible properties of Trem4 and Treml6 were confirmed in the BMDMs (Fig. 5). The mRNA levels of Trem4 and Treml6 tended to be more up-regulated in BMDMs than in BMDCs in response to polyI:C (Fig. 4a vs. Fig. 5b).

### Expression of paired trem genes in tissues and splenic DC subsets

**Fig. 6:**
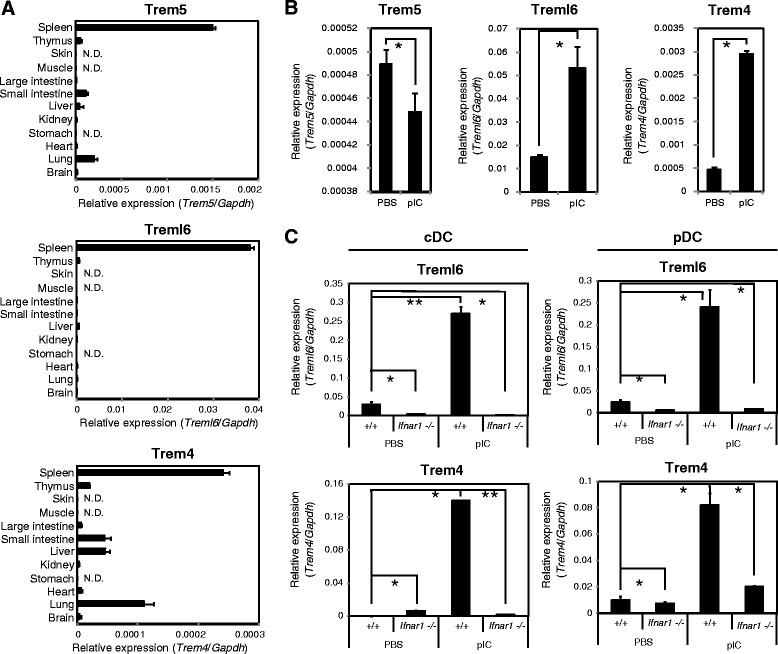
Expression of paired Trem genes in tissues and splenic DC subsets. **a** Tissue distributions of paired Trem transcripts in adult healthy mice. N.D., not detected. **b** Paired Trem expression after polyI:C stimulation in spleen. Wild-type mice were intraperitoneally injected with polyI:C (200 μg/ml). After 3 h, total RNA was isolated from spleen in each mouse. **c** Treml6 and Trem4 expression after polyI:C stimulation in splenic cDCs and pDCs. Wild-type mice were intraperitoneally injected with polyI:C (200 μg/ml). After 3 h, total RNA was isolated from FACS-sorted cDCs and pDCs. In these experiments, mRNA expressions of paired Trem genes were measured by qPCR and their expression levels were normalized to that of Gapdh mRNA. Data are representative of three independent experiments. The data shown are representative of at least two independent experiments. Data are means ± SD of three independent samples. *p < 0.05. **p < 0.01

We compared the tissue distributions of paired Trem transcripts in wild-type mice by qPCR analysis. Among the three paired Trem genes, Trem4 and Trem5 were expressed to similar levels in spleen, thymus, small intestine, liver and lung, whereas Treml6 was mainly expressed in spleen (Fig. 6a). Thus, all these paired Trem transcripts are highly expressed in the spleen, only where Treml6 ITIM-inhibitory effect can be exerted. To examine whether polyI:C-MAVS stimulation induces expression of the paired Trem genes, mice were administered with polyI:C by intraperitoneal injection, and monitored their expression levels in the spleen by qPCR analysis. In conformity with the results observed in BMDCs, polyI:C stimulation was able to induce the expression of Trem4 and Treml6, but not Trem5 (Fig. 6b). Therefore, these data indicate that expression of DAP12-associated Trem4 and Trem5 genes are differentially regulated in vivo. To investigate whether type I IFN is required for mRNA induction of Trem4 and Treml6 in splenic cDC and pDC, we monitored the expression levels of Trem4 and Treml6 mRNA in FACS-sorted cDC and pDC from wild-type and *Ifnar1*-deficient mice (Fig. 6c). We found that the mRNA expression of Trem4 and Treml6 is induced in both cDCs and pDCs in wild-type mice following polyI:C-injection, whereas their expression is abolished in *Ifnar1*-deficient mice. These results indicate that type I IFN is required for Trem4 and Treml6 induction in spleen–resident cDCs and pDCs as in BMDCs.

## Discussion

This report is the first to provide evidence that mouse has paired Trem receptors in myeloid cells and increases the expression levels in response to type I IFN. The term “paired receptor” is commonly used to describe families of membrane receptors that have similar extracellular regions but different TM and CPR. Trem4/Trem5 vs. Treml6 are typical paired receptors in the murine linage (Figs. 1 and 2b). PolyI:C stimulation triggers the mRNA expression of Trem4 and Treml6 via type I IFN, which is an output produced through the MDA5/MAVS pathway in BMDCs (Figs. 4b, c, d and 6c). All paired Trem genes are highly expressed in secondary lymphoid organs such as the spleen, which implies that the pair receptor effect coincides with an event of type I IFN induction. The Syk/SLP76-PKC or PI3K pathways are known to be activated downstream of their signal, where type I IFN also participates. Therefore, Trem4, Trem5 and Treml6 may participate in innate immune response in a myeloid-specific manner.

We also identified two novel ITIM-containing Trem genes, named as Treml7 in the dog and horse genomes and Treml8 in the rabbit and opossum genomes (Fig. 2a, b). Interestingly, either of these genes are lacking in other mammalian genomes. This finding indicates that species-specific construction occurs in paired Trem receptors. Phylogenetic analysis shows that the murine paired Trem genes are most closely related to Treml7 genes, but not Treml8. Hence, murine paired Trem genes and Treml7 are likely to originate from a common ancestral gene. A possible evolution of murine paired Trem genes is summarized in Fig. 2c. Initially, the ancestral paired Trem gene and Treml7 arose from a common ancestral gene that encoded two ITIMs in its CPR. After branching off of the murine linage, the ancestral paired Trem gene containing ITIMs was duplicated, and the murine Treml6 gene evolved from one of the duplicated genes. Another duplicated gene was translocated between the Trem1 and Foxp4 genes and lost its ITIMs. Finally, the common ancestor of Trem4 and Trem5 genes was duplicated, and evolved into murine Trem4 and Trem5.

Notably, the above-mentioned model clearly shows that activating Trem4 and Trem5 are derived from the ancestral ITIM-containing Trem gene. In human, siglec-14 or human/murine NK receptors fall into the same family [1, 37–39]. Barclay and Hatherley propose the “counterbalance theory” to explain the evolution of paired receptors [6]. In this theory, the driving force in the evolution of paired receptors is that pathogens target the inhibitory receptors on immune cells and then activating receptors evolve from these inhibitory receptors [6].

Although macrophages and DCs express Trem4, Trem5 and Treml6, natural ligands for these Trems have not been identified in either endogenous or microbial origin. How these receptors interact with NK cells, therefore, remains undetermined. Trem proteins other than Trem4/Treml6 are involved in inflammatory response [26]. Trem1 is a pro-inflammatory mediator that amplifies endotoxin-induced shock in sepsis [17, 40]. Trem1 constitutively associates with DAP12 and recognizes endogenous ligands, which is indispensable for induction of intracellular signals [17, 41]. The Trem1 signal appears to act in concert with other pattern-recognition receptors (PRRs) including TLRs and modify the cytokine/chemokine profile in myeloid cells, but its physiological mechanism remains largely elusive. Trem2 also couples with DAP12, and make a crosstalk with TLR signaling [42]. Trem2 essentially (but not simply) suppresses inflammatory responses, although their endogenous ligands are largely unknown [26, 41, 42]. Trem1 and Trem2 appear differentially work in the context of inflammation and microbial environment.

Watarai et al. showed that the Trem4-DAP12 complex mediates positive signaling for type I IFN production in Flt3 ligand-induced pDCs [25]. Here we find that direct association of DAP12 with Trem4 and Trem5 is required for their post-translational modification and cell surface expression (Fig. 3). Both receptors may lead to activation of Src-family kinases and subsequent phosphorylation of paired tyrosine residues in the ITAM of DAP12. On the other hand, activation of the MAVS pathway and type I IFN are required for surface-expression of Trem4. PolyI:C induce high surface-expression of Trem4 in the presence of DAP12, while polyI:C alone sustains high expression of Treml6 independent of DAP12. Notably, none of the TLR agonists tested participate in their expression. There is a gene-inducing program in DCs that evokes DC priming in response to internalization of viral RNA [43], where DAP12-associated Trem4 induction would be involved. Since infected DCs usually induce cell death [44], viral RNA is likely to be extrinsically taken up into DC/macrophages in the form of exosome or debris. What is responsible for Trem5 expression in combination with DAP12 is unknown. Trem5 expression might be controlled by other innate receptors such as C-type lectin receptors and NOD-like receptors.

BMDMs more efficently induce up-regulation of Trem4 and Treml6 in response to polyI:C compared to BMDCs (Fig. 4a vs. Fig. 5b). Trem-DAP12 complex may facilitate macrophage activation by enhancing phagocytosis and cytokine production. In fact, the Trem2-DAP12 axis plays a critical role in evoking phagocytosis in addition to production of inflammatory cytokines in macrophages and granulocytes in mice challenged with LPS [45]. Taken together with our results that both Trem4 and Treml6 are expressed through type I IFN signaling in macrophages and DC subsets, Trem proteins may be engaged in fine-tuning of DC maturation and myeloid immune response via the DAP12 axis and IFN-inducing pathways. The repertoire of Trem receptors has been evolutionally selected depending on species and environment. In mouse, Trem proteins modify allergic states and host response to some infections [46, 47], which will be etiologically connected with the profiles of the Trem expression.

### Ethics approval

This research does not involve human materials. All animal research protocols for this work were reviewed and approved by the Animal Safety Center, Hokkaido University, Japan.

### Availability of data and materials

The dataset supporting the conclusions of this article is included within the article.

## Additional file

Additional file 1: Table S1. Ensembl gene ID of Trems in vertebrates. (DOC 46 kb)

## Abbreviations

BM: bone marrow
DAP: DNAX activating protein
DC: dendritic cell
FlaA: flagellin A
IFN: interferon
IFNAR: IFN alpha receptor
ITAM: immunoreceptor tyrosine-based activation motif
ITIM: immunoreceptor tyrosine-based inhibition motif
MAVS: mitochondrial antiviral signaling protein
NK: natural killer
pDC: plasmacytoid DC
polyI:C: polyinosinic-polycytidylic acid
PRR: pattern-recognition receptors
TICAM: Toll-IL-1 receptor domain-containing adaptor molecule
TLR: Toll-like receptor
TM: transmembrane
Trem: triggering receptors expressed on myeloid cells
