# Supplementary material for: Double-stranded RNA analog and type I interferon regulate expression of Trem paired receptors in murine myeloid cells
Source: BMC Immunol. 2016 May 3;17:9. doi: 10.1186/s12865-016-0147-y (PMC4855714; doi:10.1186/s12865-016-0147-y)
Supplement: Additional file 1: Table S1. — Ensembl gene ID of Trems in vertebrates. (DOC 46 kb) [file 12865_2016_147_MOESM1_ESM.doc]

Additional file 1: Table S1 Ensembl gene ID of Trems in vertebrates.

|  | Humans^*^ | Mice^*^ | Rabbits^*^ | Dogs^*^ | Horses^*^ | Cows^*^ | Pigs^*^ | Opossums^*^ | Chickens^*^ | Frogs^*^ |
| --- | --- | --- | --- | --- | --- | --- | --- | --- | --- | --- |
| Trem1 | 124731 | 42265 | 11397 | 1591 | 17436 | 17593 | 1617 | — | — | — |
| Trem2 (Trema) | 095970 | 23992 | 07265 | 1586 | 15270 | 07275 | 1614 | 13004 | 23781 | 32041 |
| Trem3 | — | 41754 | 23700 | — | — | — | — | 28376 (Trem3.1) | — | — |
|  |  |  |  |  |  |  |  | 12942 (Trem3.2) |  |  |
| Treml1 | 161911 | 23993 | 07264 | 1584 | 12615 | 06485 | 1613 | 13007 | — | — |
| Treml2 | 112195 | 71068 | 07268 | 1589 | 16341 | 15707 | 1616 | 29432 | — | — |
| Treml4 | 188056 | 51682 | — | — | — | — | — | — | — | — |
| Trem5 | — | 73386 | — | — | — | — | — | — | — | — |
| Treml6 | — | 43740 | — | — | — | — | — | — | — | — |
| Trem4 | — | 43939 | — | — | — | — | — | — | — | — |
| Treml7 | — | — | — | 1587 | 15483 | — | — | — | — | — |
| Treml8 | — | — | 25716 | — | — | — | — | 28475 | — | — |
| Tremb1 | — | — | — | — | — | — | — | — | 03361 | — |
| Tremb2 | — | — | — | — | — | — | — | — | 23780 | — |

^*^ENSG00000, ENSMUSG000000, ENSOCUG000000, ENSCAFG0000000, ENSECAG000000, ENSBTAG000000, ENSSSCG0000000, ENSMODG000000, ENSGALG000000 and ENSXETG000000 were abbreviated from the front of human, mouse, rabbit, dog, horse, cow, pig, opossum, chicken and frog Ensembl gene ID numbers, respectively (e.g., 124731 in this table means ENSG00000124731). In Ensembl genome browser, murine Trem5, Treml6 and pDC-Trem are labeled as 9830107B12Rik, B430306N03 and A530064D06Rik, respectively. Similarly, rabbit Trem3 and Treml7 are labeled as ENSOCUG00000023700 and ENSOCUG00000025716, respectively. Dog Treml7 is labeled as ENSCAFG00000001587. Horse Treml7 is labeled as ENSECAG00000015483. Opossum Treml8, Trem3.1 and Tren3.2 are labeled as ENSMODG00000028475, ENSMODG00000028376 and ENSMODG00000012942, respectively. —: not found.
